# Umbilical mesenchymal stem cell-derived extracellular vesicles as enzyme delivery vehicle to treat Morquio A fibroblasts

**DOI:** 10.1186/s13287-021-02355-0

**Published:** 2021-05-06

**Authors:** Michael Flanagan, Isha Pathak, Qi Gan, Linda Winter, Ryan Emnet, Salem Akel, Adriana M. Montaño

**Affiliations:** 1 Department of Pediatrics, School of Medicine, Saint Louis University, 1100 South Grand Blvd., Room 313, St. Louis, MO 63104 USA; 2 School of Medicine, Saint Louis University, Saint Louis, Missouri USA; 3 St. Louis Cord Blood Bank, SSM Cardinal Glennon Children’s Medical Center, St Louis, MO USA; 4 Department of Biochemistry and Molecular Biology, School of Medicine, Saint Louis University, Saint Louis, Missouri USA

**Keywords:** Umbilical mesenchymal stem cell, Extracellular vesicles, Mucopolysaccharidosis IVA, Morquio A

## Abstract

**Background:**

Mucopolysaccharidosis IVA (Morquio A syndrome) is a lysosomal storage disease caused by the deficiency of enzyme *N*-acetylgalactosamine-6-sulfate sulfatase (GALNS), which results in the accumulation of the glycosaminoglycans (GAGs), keratan sulfate, and chondroitin-6-sulfate in the lysosomes of all tissues causing systemic dysfunction. Current treatments include enzyme replacement therapy (ERT) which can treat only certain aspects of the disease such as endurance-related biological endpoints. A key challenge in ERT is ineffective enzyme uptake in avascular tissues, which makes the treatment of the corneal, cartilage, and heart valvular tissue difficult. The aim of this study was to culture human umbilical mesenchymal stem cells (UMSC), demonstrate presence of GALNS enzyme activity within the extracellular vesicles (EVs) derived from these UMSC, and study how these secreted EVs are taken up by GALNS-deficient cells and used by the deficient cell’s lysosomes.

**Methods:**

We obtained and cultured UMSC from the umbilical cord tissue from anonymous donors from the Saint Louis Cord Blood Bank. We characterized UMSC cell surface markers to confirm phenotype by cell sorting analyses. In addition, we confirmed that UMSC secrete GALNS enzyme creating conditioned media for co-culture experiments with GALNS deficient cells. Lastly, we isolated EVs derived from UMSC by ultracentrifugation to confirm source of GALNS enzyme.

**Results:**

Co-culture and confocal microscopy experiments indicated that the lysosomal content from UMSC migrated to deficient cells as evidenced by the peak signal intensity occurring at 15 min. EVs released by UMSC were characterized indicating that the EVs contained the active GALNS enzyme. Uptake of GALNS within EVs by deficient fibroblasts was not affected by mannose-6-phosphate (M6P) inhibition, suggesting that EV uptake by these fibroblasts is gradual and might be mediated by a different means than the M6P receptor.

**Conclusions:**

UMSC can deliver EVs containing functional GALNS enzyme to deficient cells. This enzyme delivery method, which was unaffected by M6P inhibition, can function as a novel technique for reducing GAG accumulation in cells in avascular tissues, thereby providing a potential treatment option for Morquio A syndrome.

## Background

Mucopolysaccharidosis IVA (MPS IVA, Morquio A syndrome) is an autosomal recessive disorder characterized by the deficiency of the lysosomal enzyme *N*-acetylgalactosamine-6-sulfate sulfatase (GALNS). Deficiency of this enzyme results in accumulation of the glycosaminoglycans (GAGs), keratan sulfate (KS), and chondroitin-6-sulfate (C6S) in lysosomes of tissues [1]. The presentation of symptoms in Morquio A varies based on the severity of the disorder. The accumulation of GAGs results in skeletal dysplasia involving short stature, odontoid hypoplasia, scoliosis, kyphosis, *genu valgum*, joint laxity, chest deformity including *pectus carinatum*, and rib cage flaring. Other manifestations include clouding of the corneas, valvular disease of the heart [2], and increased carotid intra-media thickness [3], which is a potential indicator of atherosclerosis [4]. Those with a milder phenotype present with fewer clinical manifestations and a longer life span [5–9]. Often Morquio A patients undergo corrective orthopedic surgeries for the neck, hip, and leg region, including cervical spine fusion, corrective knee surgery for the knock-knee deformity, and femoral or tibial osteotomies for straightening the legs. These complications and surgeries often result in those patients with severe phenotype not surviving beyond the second or third decade of life [1, 5].

Traditionally, treatment of Morquio A syndrome has been limited to management of symptoms and palliative care; however, enzyme replacement therapy (ERT) using recombinant human GALNS enzyme (rhGALNS) has shown great advancements and promise. ERT involves treating the patient by replacing the missing or deficient enzyme with an intravenous infusion of the recombinant enzyme [10]. Currently, Elosulfase alfa (Vimizim®) is the ERT available for Morquio A syndrome, which received FDA approval in 2014 [11]. Clinical trials have shown that in some Morquio A patients the use of Elosulfase alfa has maintained or improved the levels of certain biological endpoints, such as the 6-min walk test [12, 13], which is in contrast to the decline observed in the natural history of Morquio A [1, 14].

The use of ERT as a treatment for MPS disorders is not without its shortcomings and challenges. While Elosulfase alfa does show some improvement in patients’ endurance markers, challenges, and limitations still exist with ERT for Morquio A syndrome. During clinical trials, a small number of patients experienced adverse reactions to ERT. Sixty percent of patients experienced at least one hypersensitivity reaction, such as angioedema or an anaphylactic reaction, and the most frequent reactions were pyrexia or headaches. In addition, all patients that underwent ERT developed antibodies to Elosulfase alfa [12]. This presents a strong challenge for ERT over time, as Morquio A patients need immunosuppressors to prolong its efficacy. One such regimen incorporated a pre-treatment with antihistamines and antipyretics prior to infusion [15]. Recently, we demonstrated induced immunosuppression in vivo by orally administering rhGALNS or immunodominant GALNS peptides prior to ERT. The study demonstrated that oral tolerance induced a reduction in both the humoral and cellular response to the GALNS enzyme, increasing ERT efficacy [16].

A key challenge of ERT is the limited enzyme uptake in avascular tissues, which makes it very difficult to treat disease manifestations in the cornea, cartilage, and heart valvular tissues [17, 18]. Earlier experiments found that using intravenous rhGALNS ERT to treat Morquio A mice not only showed little improvement in cartilaginous tissues but also rapid clearance of enzyme from the blood [19].

Stem cell therapy has been used to treat a wide number of conditions, including MPS disorders [20]. Hematopoietic stem cells therapy has previously been used for Morquio A in Japanese patients [2, 21, 22]. The first of these studies followed a patient who received bone marrow transplant (BMT) to supplement the normal regimen of ERT. The study showed that, over the course of 9 years, the patient’s white blood cells maintained a GALNS activity level of approximately 50% of normal non-Morquio A individuals [21]. However, these treatments present a set of limitations, such as increased risk of mortality, which is mainly due to complications from the procedure or from graft vs host disease. Previous attempts at BMT were unsuccessful, in part due to being performed on terminal Morquio A patients [22]. In a more recent study, BMT was performed at a younger age and showed improvements in several phenotypical conditions, such as improved walking and reduced skeletal dysplasia [22].

In addition to bone marrow [23], mesenchymal stem cells (MSC) can be harvested from a variety of different tissues. These tissues include endothelial tissue [24], adipose tissue [25, 26], umbilical tissue [27], or even smaller reservoirs such as the follicle or pulp of unerupted teeth [28, 29]. MSC isolated from the umbilical cord, hereby termed umbilical mesenchymal stem cells (UMSC), are of unique interest to this study. UMSC present an attractive treatment option for many reasons, such as ease of acquisition, availability, and pluripotency. Unlike MSCs derived from the bone marrow or adipose tissue, the umbilical tissue is easily obtained through non-invasive means and would otherwise be discarded as medical waste [30].

As with other MSCs, UMSC have the ability to release extracellular vesicles (EVs) [31], which contain therapeutic payloads that can facilitate the repair of local tissues [31, 32]. These EVs are heterogeneous in size and composition, but can be generally classified into two groups: exosomes and microvesicles. Exosomes are small vesicles, usually 150nm in diameter or less, that form within the multi-vesicular endosomes and are released by exocytosis when the multi-vesicular endosome fuses with the outer plasma membrane [33–35]. By contrast, microvesicles are formed from the plasma membrane itself, which buds off and detaches. As such, they are considerably larger than exosomes, with most having a diameter of 200nm or greater [35]. The use of EVs has been reported in a variety of treatments, including Parkinson’s disease [36], kidney repair [32], fibrosis of the liver [37], and myocardial infarction [38]. EVs are immunomodulatory, and studies have shown that large amounts of EVs are present in the tumor microenvironment [39]. More interestingly, EVs themselves do not induce an immune response [40], and there is mounting evidence that treatment with MSC-derived EVs can perform the therapeutic functions of MSC without directly transplanting cells into the target tissues [41]. MSC-derived EVs have recently been used to treat lysosomal storage diseases [42, 43], and improvements in the corneas in MPS VII models have been demonstrated [44, 45]. Given the avascular nature of the cornea, the use of MSC-derived EVs could present a potentially novel treatment option in Morquio A patients.

In this study, we hypothesize that UMSC can restore enzyme function in GALNS-deficient cells through the release of EVs. To this end, we cultured UMSC and demonstrated their ability to secrete active GALNS enzyme, as well as EVs containing GALNS. We have also demonstrated that these secretions are taken up by GALNS-deficient cells in an active form and used within the cell’s own lysosomes.

## Methods

### Human samples

Anonymous umbilical mesenchymal stem cells were donated by the St. Louis Cord Blood Bank, where they were isolated and described previously [46, 47]. All UMSC in this study were isolated by collagenase digestion and displayed the capability to differentiate into osteogenic, osteogenic, and adipogenic lineages [46], which are known features of UMSC [48, 49]. Morquio A fibroblasts were obtained from a de-identified repository located at Saint Louis University. The Institutional Review Board (IRB) at Saint Louis University determined that our human subjects research was exempt from a formal IRB submission due to a lack of patient identifiers or protected health information (PHI).

### Cell culture

Unless otherwise specified, cells were seeded to an initial density of 2000 cells/cm^2^. Cells were grown in either MEM-α supplemented with 15% fetal bovine serum or in MesenPRO RS Mesenchymal Stem Cell media (Gibco, New York, USA). Media was changed by replacing half the volume every other day. At 80% confluence, cells were harvested via trypsinization and re-seeded at the above density to fresh plates for further expansion [46, 49]. Only stem cells between passages 2 and 10 were used for experimentation.

### UMSC surface marker phenotyping

We measured cell surface markers using the MACS MSC Phenotyping Kit (Miltenyi Biotec, Bergisch Gladbach, Germany) that measure expression of CD 90, CD73, and CD105, which are the accepted standard markers of MSC [50]. Briefly, two aliquots of 1.0 × 10^6^ UMSC were suspended in 100μL culture media. 10 μL of MSC phenotyping cocktail was added to one suspension; 10 μL of isotype control was added to the second. Cells were labeled in the dark at 4°C for 10 min before rinsing with PBS and measured by FACS sorting. FACS sorting was performed on a FACSCanto II, using BD FACSDiva Software v6 (BD Biosciences, New Jersey, USA). Visualization of these results was formed using FlowJo v10 (FlowJo LLC, Oregon, USA).

### Measurement of cellular GALNS activity

Cells were lysed using 50–100μL of 1% sodium deoxycholate solution (Sigma, Missouri, USA). The primary substrate used for GALNS enzyme activity assay was a 22-mM solution of 4-methylumbelliferyl β-d-galactopyranoside-6-sulfate (4-MU Gal-6-S) [51]. GALNS activity was measured as reported elsewhere [1]. Briefly, samples were incubated with 4-MU Gal-6-S at 37°C, for 15 min. Next, a secondary incubation with 10-mM β-galactosidase at 37°C for 30 min was performed prior to fluorometric analysis [51, 52]. All cellular enzyme activity was normalized to protein concentration of the lysate.

### Conditioned media

UMSC were grown using cell culture methods described previously. Once 70% confluence was reached, the cells were grown in media which was conditioned until the cells reached 100% confluence. Media was collected and concentrated by centrifugation in a centricon device (Millipore, Massachusetts, USA) at a size of 30,000 NMWL at 3000rpm at 4°C for 15–20 min and immediately used for treatment [1].

### Mannose-6-phosphate-mediated uptake

To test the mediation of GALNS uptake in UMSC, cells were seeded to a 12-well plate at a density of 5.0×10^4^ cells/well. For all cells, media was supplemented with 2000 units GALNS/mL. Experimental groups were supplemented with 2-mM mannose-6-phosphate. Cells were incubated for 5h or 24h before lysis and measurement of enzyme activity [1].

### Co-culture of UMSC with deficient fibroblasts

For co-culture, 2.0 × 10^5^ fibroblasts/well were each seeded to 12-well plates. UMSC were seeded to the interior of 1.0-μm PET transwell inserts (Millipore, Massachusetts, USA) at a density of 3.4×10^4^ cells/insert. When co-culture was performed for confocal microscopy, cells were grown in cover glass 12-well plates or seeded to co-culture slides (Ibidi, Bavaria, Germany). For concentric co-culture slides, the outer 8 surrounding minor wells were seeded with feeder cells (UMSC), with the recipient cells (Morquio A deficient fibroblast) seeded to the central minor well. All cells were seeded at a density of 7.0×10^3^ cells/minor well [53].

### Isolation of extracellular vesicles from UMSC

Extracellular vesicles (EVs) were isolated by differential ultracentrifugation at 4°C, as described by Li et al. [54]. Briefly, UMSC were grown to 70% confluence. Cells were grown in serum-free media, which was conditioned as cells grew from 70 to 100% confluence. Conditioned media was centrifuged at 300rcf to pellet any loose cells from the media. Next, the supernatant was collected and spun down at 10,000rcf to pellet any remaining cell debris. The supernatant was again collected and sterile filtered through a 0.22-μm membrane. The filtrate was then spun down via ultracentrifugation at 100,000rcf for 90 min, and the supernatant was removed. Pellet was resuspended in PBS at 1:1000 volume of the original filtrate.

### Cellular organelle labeling

Cells were seeded 1 day prior to co-culture. Immediately before co-culture, UMSC were stained with Lysotracker Red DND-99 (Invitrogen, California, USA). Briefly, the culture media was removed from the cells and replaced with MesenPRO RS containing 50-nM lysotracker dye. All non-stained cells received fresh media. Cells were incubated at 37°C for 1h. Next, cells were washed three times with fresh culture media prior to co-culture. Hoechst 33342 nuclear stain was done using NucBlue Live Cell Stain (Thermofisher, Massachusetts, USA) immediately before imaging, as recommended by the vendor [44].

### Confocal microscopy

Co-cultured cells were imaged on a Leica SP8 TCS STED 3X. Cells were imaged at ×50 magnification every 5 min over a period of 150 min. Lysotracker red fluorescence and nuclear staining were measured. Images were collected using the Leica LAS X analysis software (Leica Microsystems, Wetzlar, Germany). Images were exported and recompiled in ImageJ software [55].

### Enzyme collection and purification

Enzyme was collected from CHO cells modified to constitutively express active rhGALNS and then purified as described previously [1, 52]. Briefly, CHO cells were grown to confluence before culturing in Ex-Cell Serum Free Media (Sigma, Missouri, USA). Cell media was collected, dialyzed, concentrated, and purified by ion exchange (CM sepharose) and size exclusion (S-300 and S-100 columns) chromatography. Purity was confirmed by SDS-PAGE and measuring specific activity. Pure enzyme fractions were concentrated, and the final product was aliquoted and stored at −80°C.

### Western blot analysis

Extracellular vesicles were isolated as described above. EVs were lysed in 1% sodium deoxycholate solution. 125μg of sample lysate was loaded onto a 12.5% SDS-polyacrylamide gel. Next, samples were blotted to a PVDF membrane at 100V for 90 min at 4°C. The membrane was blocked in TBST solution containing 5% dry milk for 1 h at RT before probing with antibodies overnight at 4°C [56]. Primary antibodies used were a mouse α-actin (Cell Signaling Technology, Massachusetts, USA) at a 1:1000 dilution and a mouse α-GALNS at 1:1,000,000 in 5% dry milk solution [57]. Both primary antibodies were probed with the same secondary antibody, an HRP-conjugated goat α-mouse IgG (Cell Signaling Technology, Massachusetts, USA) for 1 h at RT.

### Statistical analysis

Statistical analysis was performed in SPSS 23 (IBM SPSS Inc., Chicago, USA). Unless otherwise noted, treatments were conducted in triplicate. Measurement of each treatment was also measured in triplicate. All enzyme activity data collected from cellular lysates were normalized by protein concentration. Means were compared by independent 2-tailed unpaired *t* test with Welch’s correction. Significance was defined as a *p* value less than 0.05.

## Results

### UMSC surface markers exhibit MSC phenotype

**Fig. 1:**
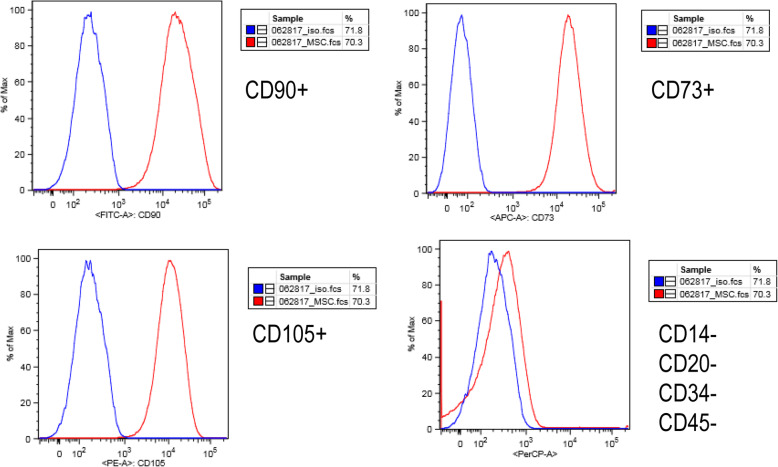
Surface marker expression confirms the mesenchymal phenotype of UMSC. UMSC were labeled with fluorescent-tagged antibodies (red) and compared to unlabeled controls (blue). A rightward shift in peaks indicates the presence of the stained markers. UMSC expressed the markers CD90 (**a**), CD73 (**b**), and CD105 (**c**). The lack of the rightward shift in (**d**) indicated that UMSC do not display the markers CD14, CD20, CD34, and CD45

Given the nonselective nature of MSC isolation and the heterogeneity of isolated populations [58], we confirmed that the UMSC possessed the mesenchymal phenotype. We first labeled the cells with markers specific to different stem cell types. The cells displayed the stem cell markers CD90, CD73, and CD105 (Fig. 1a–c). In contrast, the cells did not display the hematopoietic stem cell markers CD14, CD20, CD34, or CD45 (Fig. 1d). These results suggest that the cells in culture are adult, somatic stem cells and that they do not have a hematopoietic stem cell phenotype. This evidence indicates that the cells in culture are mesenchymal stem cells.

### Inhibition of GALNS uptake by mannose-6-phosphate

**Fig. 2:**
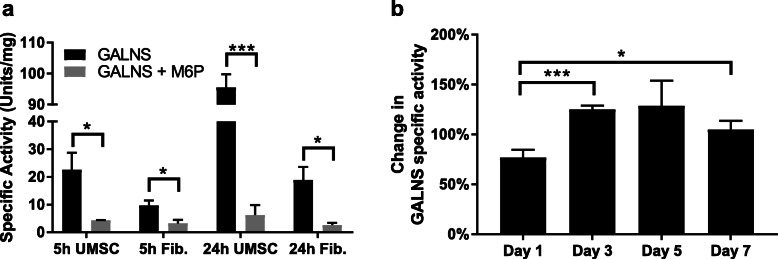
UMSC are capable of uptake and release GALNS enzyme. **a** Specific activity of UMSC and deficient fibroblast lysate when cultured in rhGALNS-supplemented media. Both cells showed a strong increase in GALNS activity from 5h to 24h. After the addition of mannose-6-phosphate, both cells showed a decrease in activity, with UMSC showing an 80–90% reduction, and the fibroblasts having a 65–85% reduction after 24 h. **b** Specific activity of fibroblasts co-cultured with UMSC, normalized to fibroblasts cultured without UMSC. After an initial delay, the fibroblasts displayed a 25% increase in GALNS activity on days 3 and 5, then returning to baseline on day 7. Error bars: mean ± SD. (*P < 0.05, ***P < 0.001, two-tailed unpaired *t* test with Welch’s correction)

We tested the ability for UMSC to uptake rhGALNS when compared to GALNS-deficient fibroblasts. We cultured both UMSC and GALNS-deficient fibroblasts using rhGALNS-supplemented media. To evaluate whether rhGALNS uptake was facilitated via the mannose-6-phosphate (M6P) receptor [1, 59], we also cultured both UMSC and deficient fibroblasts in the presence or absence of M6P. After 5 or 24 h of culture, we observed a fourfold increase in GALNS uptake in UMSC, compared to the twofold increase in fibroblast uptake. In addition, we found that M6P inhibited the uptake by UMSC by 95% (Fig. 2a). Next, we co-cultured UMSC with deficient fibroblasts to determine if the fibroblasts could uptake the GALNS released from the UMSC into the culture media, as shown in (Fig. 2b). The specific activity of treated fibroblasts was normalized to untreated ones. Initially, co-cultured cells showed reduced activity. However, co-cultured cells had 25% more activity than in untreated fibroblasts by days 3 and 5. This increased activity returned to baseline by 7 days. Based on these observations, we conclude that co-culturing deficient cells with UMSC displays improved GALNS activity.

### Transfer of lysosomes from UMSC to deficient cells in vitro

**Fig. 3:**
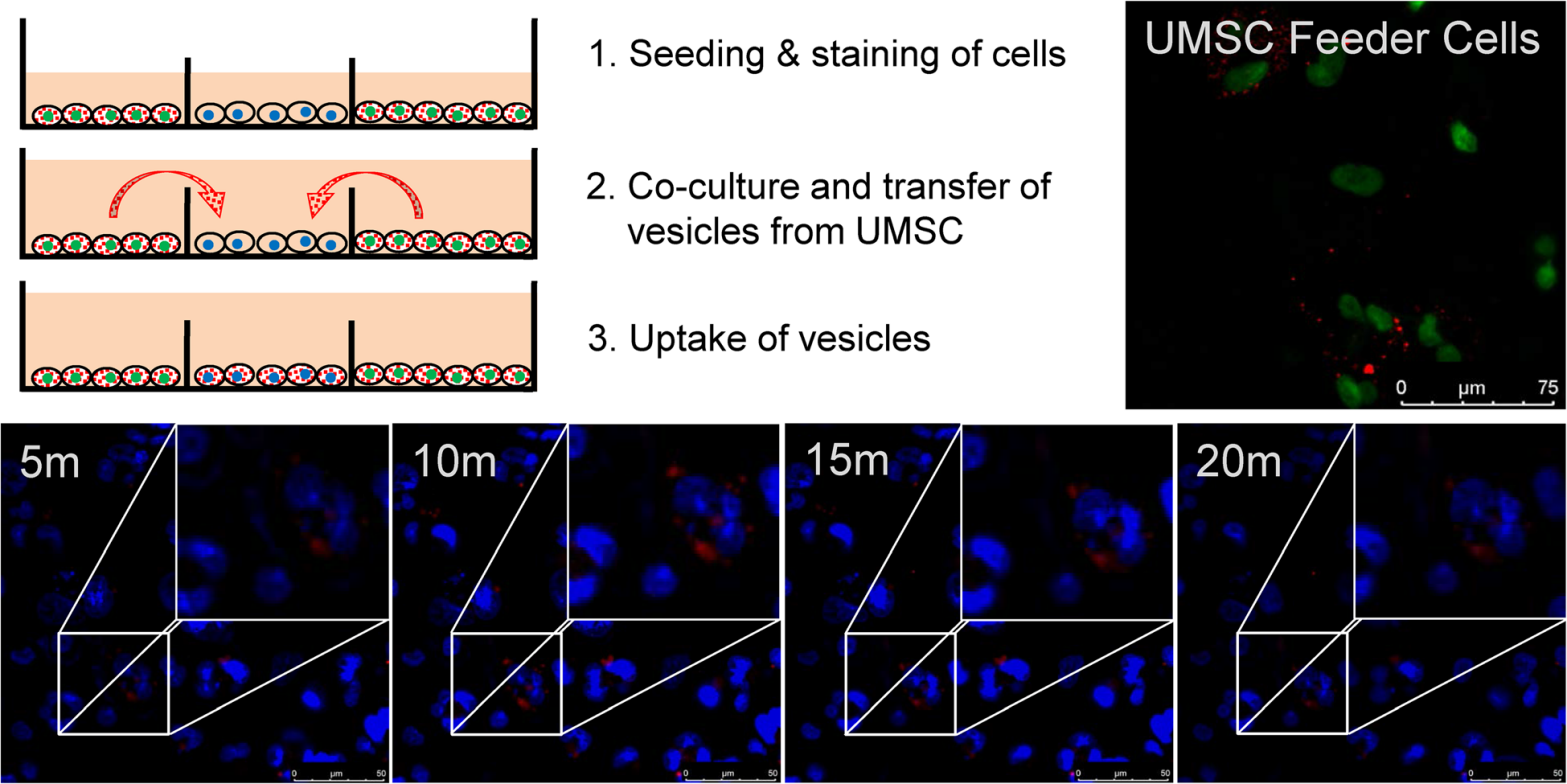
Co-culture of UMSC reveals transfer of material to deficient cells. Lysosomes (red) originating from Hoechst labeled UMSC feeder cells (green) were fluorescently labeled with Lysotracker Red prior to co-culture with Hoechst labeled fibroblasts (blue). This signal gradually increases within the fibroblasts during co-culture with the stained feeder cells, reaching a maximum at 15 min, before gradually declining at 20 min

To confirm the movement of lysosomal content from UMSC to fibroblasts, we labeled UMSC with lysotracker red, which targets low pH organelles such as lysosomes. After labeling UMSC, we co-cultured them with deficient fibroblasts and monitored transfer of lysotracker-positive organelles from UMSC to deficient fibroblasts. We observed that the positive-labeled UMSC—here labeled as “feeder cells”—showed a strong lysotracker presence prior to co-culture. Within 10 min of co-culture, UMSC-derived lysosomes had migrated and emerged within the deficient fibroblasts. These lysosomes rapidly accumulated within the deficient cells, peaking in intensity at 15 min, before the signal began to decrease after 20 min of co-culture (Fig. 3).

### UMSC-derived extracellular vesicles contain active GALNS enzyme

**Fig. 4:**
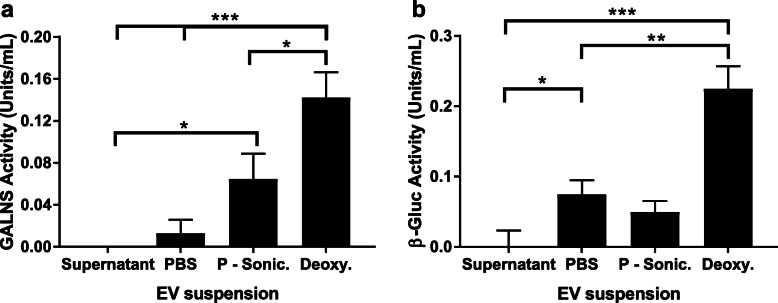
Isolated EVs contain latent enzyme. After isolating at pelleting EVs from the host UMSC, the pellet was resuspended, and the suspension was measured for GALNS (**a**) or GUSB (**b**) activity. The EV-free supernatant of the isolation process contained no detectable GALNS or GUSB activities. Suspending the EVs in PBS contained a small amount of available GALNS and GUSB. Mechanical disruption by sonication increased the amount of available active GALNS enzyme. Finally, detergent-based lysis of EVs showed a significant increase in available active enzyme for both GALNS and GUSB. Error bars: mean ± SD. (*P < 0.05, **P < 0.01, ***P < 0.001, two-tailed unpaired *t* test with Welch’s correction)

To identify the mechanism through which the GALNS enzyme is delivered from UMSC to deficient fibroblasts, as well as to prove the transmission of cellular material, we isolated and characterized the UMSC-derived extracellular vesicles (EVs), whose release had been previously observed in treating other disease models [44]. After the isolation of EVs, we measured the enzyme activity of two lysosomal enzymes GALNS and β-glucuronidase (GUSB). We detected GALNS activity in the EV isolate, and we observed high levels of GALNS activity after the EVs were lysed in a detergent based solution (i.e., deoxycholate) when compared to cells lysed via mechanical disruption (i.e., sonication) (Fig. 4a). This suggested that the latent GALNS was contained primarily within the EVs. We noted a similar pattern when assaying GUSB from the partially or completely lysed EVs (Fig. 4b).

### UMSC-EVs contain a heterogeneous mixture of microvesicles and exosomes

**Fig. 5:**
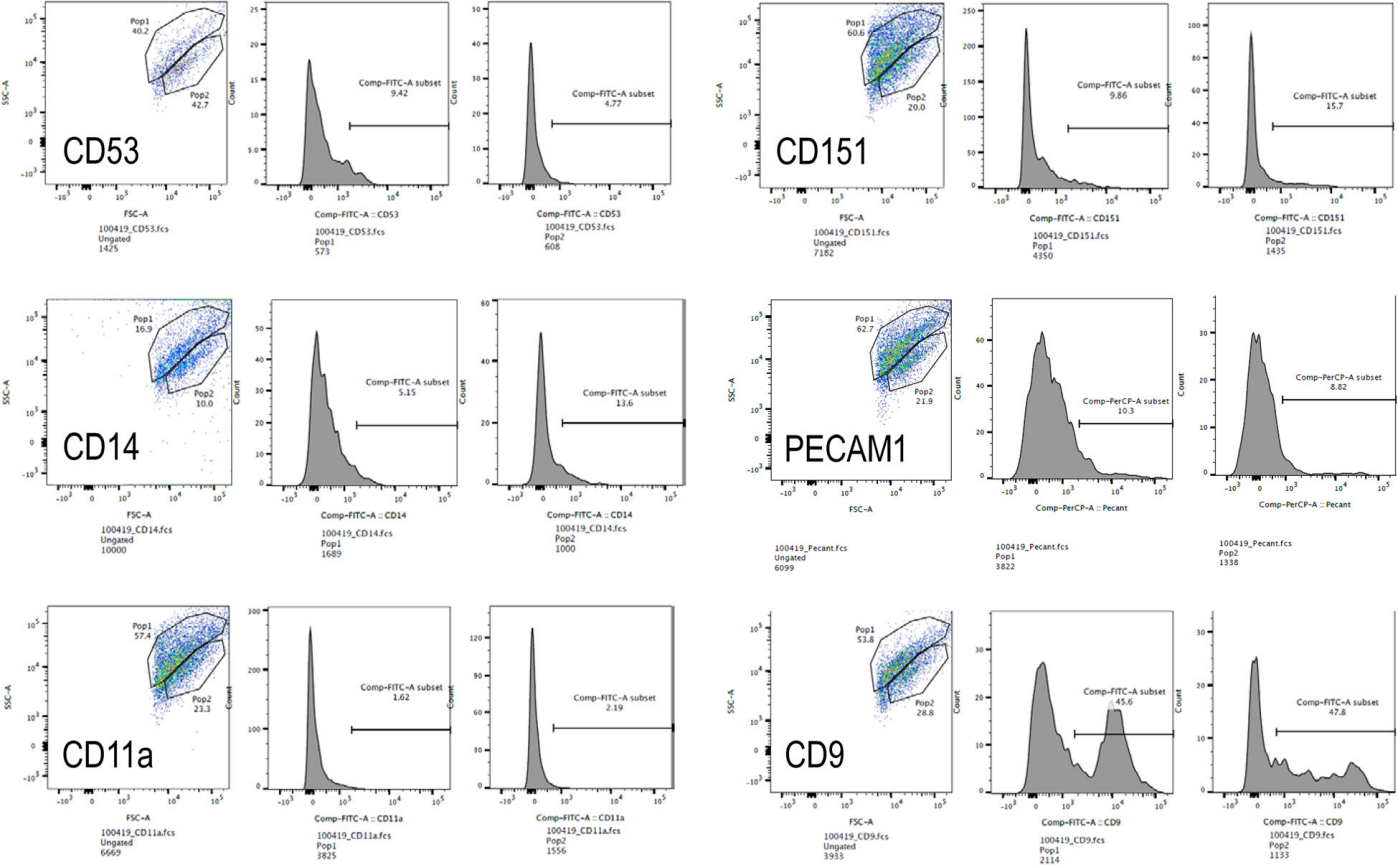
FACS scanning of EVs reveals a heterogeneous population of vesicles. UMSC-derived EVs were screened for microvesicle and exosome markers by FACS sorting. In the case of all vesicles, the EVs were separated into two populations, with the first population indicating higher side scatter than the second. Rightward peak shift indicates the presence of the stained markers. Population 1 showed a small increase in CD53+ (**a**), CD 151+ (**b**), CD14+ (**c**), and PECAM1+ vesicles (**d**). Population 2 showed a slight increase in CD151+ and CD14+ vesicles. Neither population showed an increase in CD11a+ vesicles (**e**). Finally, both populations had a strong presence in CD9+ vesicles (**f**)

EVs are heterogeneous in size and surface markers. They can be separated into two main categories: exosomes and microvesicles. Analysis of EVs by FACS sorting revealed two distinct populations when accounting for side-scatter. The high side-scatter population consisting of 48.6 ± 7.1% of all instances, while the lower side-scatter accounted for 24.5 ± 4.4% of all instances (Fig. 5). We labeled the EVs with fluorescent antibodies specific to exosomes (CD53, CD151), microvesicles (PECAM1, CD14, CD11a), or both (CD9). We then identified populations of each vesicle type by FACS scanning. The population of EVs that were isolated showed a slight rightward shift in the exosome markers CD53 and CD151 (Fig. 5a,b). There was also a marked shift in the presence of PECAM1 signaling, a microvesicle marker, with a smaller shift in CD14 (Fig. 5c,d). When we labeled the EVs with antibodies targeting antigens present in both exosomes and microvesicles, we found a similar pattern. There was no apparent shift indicating the presence of CD11a (Fig. 5e); however, we observed a significant increase in CD9+ vesicles (Fig. 5f). These results suggest that the EV isolation contains a heterogeneous mixture of both microvesicles and exosomes.

### UMSC-EVs facilitate GALNS uptake by Morquio A deficient cells

**Fig. 6:**
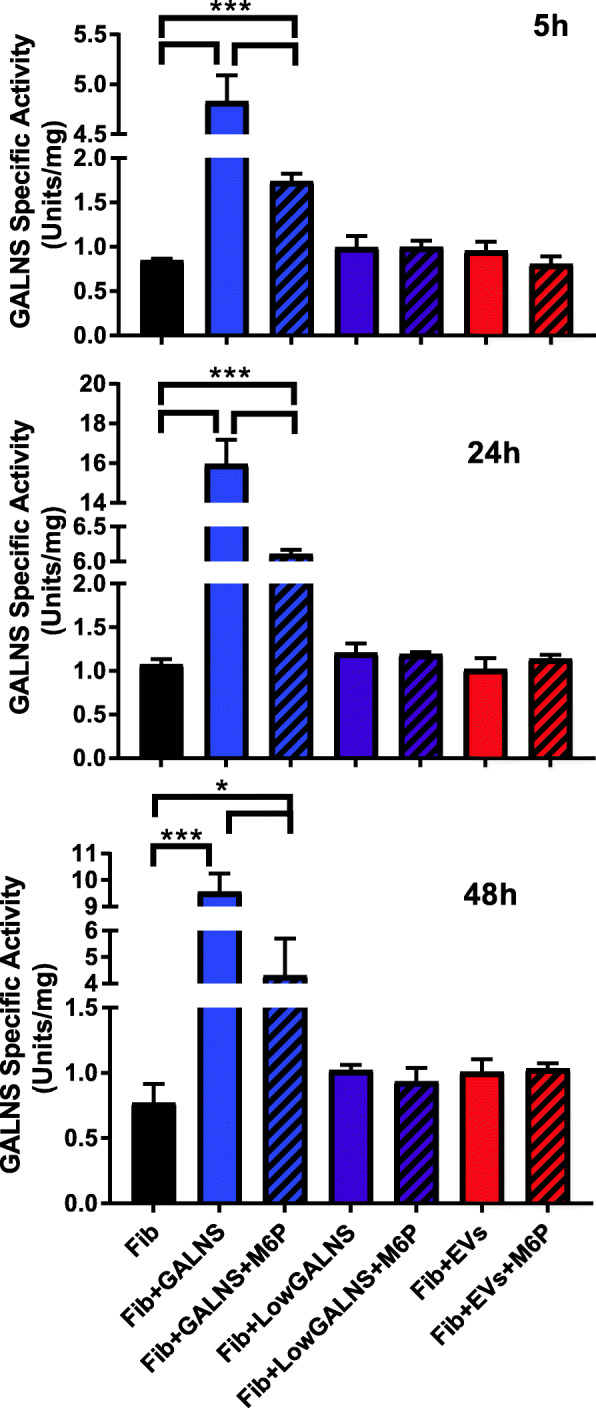
Uptake of GALNS within EVs is more gradual and unaffected by M6P inhibition. Deficient fibroblasts were cultured in 2000 U GALNS (+GALNS) in the presence or absence of M6P (blue bars). This showed a strong increase in GALNS enzyme activity through the initial 24 h before declining, and this GALNS activity was inhibited by M6P. Deficient fibroblasts cultured in GALNS with an activity equal to those found in EVs (+LowGALNS) (purple bars) as well as UMSC-derived EVs (+EVs) (red bars) had a GALNS activity increase, though not significant at 24 h. Treatment of Morquio A deficient fibroblasts with UMSC-derived EVs or small amounts of GALNS showed steady slight increase up to 48 h, and this increase was not inhibited by M6P, suggesting a more gradual uptake. Deficient Morquio A fibroblasts without addition of exogenous GALNS (black bars). Error bars: mean ± SD. (*P < 0.05, ***P < 0.001, two-tailed unpaired *t* test with Welch’s correction)

We next tested the ability for Morquio A deficient fibroblasts to uptake the latent GALNS found within EVs. While we had shown that GALNS contained within isolated EVs included active GALNS enzyme, we still needed to determine if it could be delivered to deficient cells. In addition, we checked whether M6P receptor plays a role in the EV uptake or not. We observed a significant uptake of purified rhGALNS when supplemented into culture medium at high concentrations reaching a peak at 24h. This uptake was reduced by M6P competitively inhibiting the binding of GALNS to the M6P receptor at 5h, 24h, and 48h (Fig. 6).

After 5h, 24h, and 48h of treatment with UMSC-EVs, we found a subtle increase of GALNS uptake by deficient cells, which was comparable to the values from cells treated with low concentrations of rhGALNS enzyme. After administration of M6P, there was no noticeable change in the levels of the uptaken enzyme indicating that there was no inhibition by M6P. This can be explained by the fact that there is no saturation of the M6PR due to the low and gradual amounts of GALNS enzyme that was uptaken or that the EVs containing GALNS are uptaken by a different means than the M6P receptor (Fig. 6).

### UMSC release of GALNS-carrying EVs across multiple passages in vitro

**Fig. 7:**
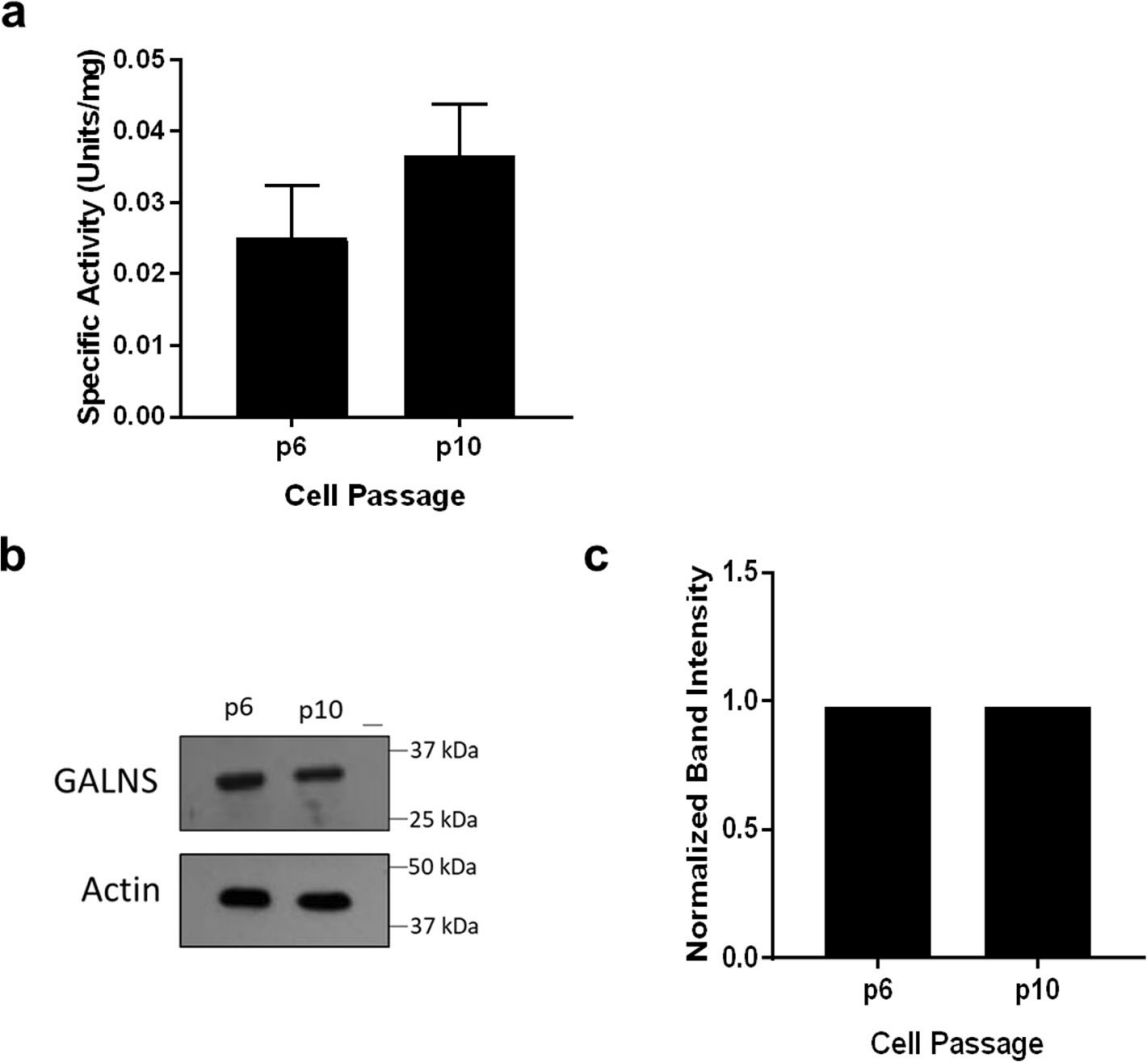
Isolation of EVs from higher-passage UMSC does not yield reduced GALNS activity. EVs were isolated from p6 and p10 UMSC, lysed, and measured for protein-normalized activity. The change in activity from p6 to p10 was not statistically significant (*P*=0.33) (**a**). Additionally, western blotting of lysates showed the GALNS enzyme present in both early and late passages (**b**). Signal normalized with actin showed equal intensity (**c**). Error bars: mean ± SD

We suspected that UMSC of later passages might release EVs at a different density or that EVs would have a different level of GALNS activity. We isolated EVs from high and low passage UMSC and measured the amount of active GALNS present. Our original hypothesis would be that high-passage UMSC would produce fewer EVs, or less overall GALNS enzyme, when compared to lower-passage UMSC. However, after isolation and complete lysis of the EVs from their respective cell passages, we did not observe any significant detrimental changes in GALNS activity up to passage 10. To confirm this finding, we performed western blot of the UMSC and fibroblasts lysates, using a monoclonal antibody against GALNS. We confirmed the presence of GALNS enzyme in both cells. Next, we blotted the lysate from purified EVs and tagged for GALNS. We found that, while a large amount of EV lysate was needed to detect the GALNS protein, it was still present in EV lysates from early and late passage UMSC (Fig. 7a–c).

### Stable transfection of UMSC overexpress GALNS enzyme

**Fig. 8:**
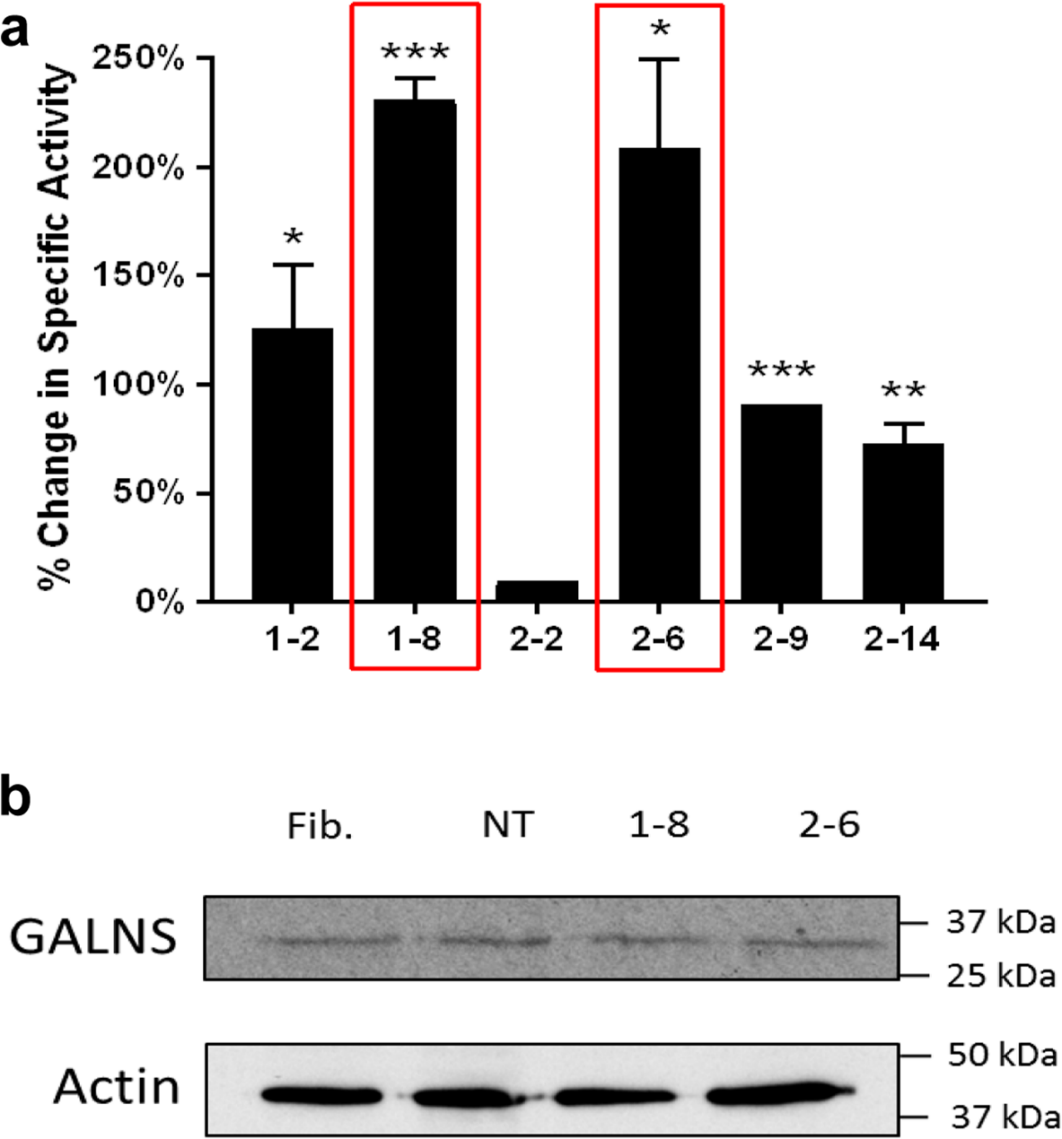
Constitutive expression of GALNS enzyme from UMSC. UMSC were transfected to constitutively express GALNS enzyme. After performing three rounds of clone selection, two clones [1–8 and 2–6] were found to express GALNS over 200% of non-transfected controls (**a**). Western blotting of these clones confirms the presence of the GALNS enzyme within these clones, as compared to non-transfected cells (**b**). Error bars: mean ± SD. (*P < 0.05, **P < 0.01, ***P < 0.001, two-tailed unpaired *t* test with Welch’s correction)

Finally, we performed stable transfection to explore the ability of UMSC to overexpress high amounts of GALNS enzyme. After three rounds of clone selection, we found two clones [1–8 and 2–6] that had over 200% higher activity than non-transfected cells (Fig. 8a,b). This finding provides a new tool that can be used in conjunction with the EVs for potential treatment.

## Discussion

Mesenchymal stem cells have been the subject of many therapeutic treatments. Of interest are UMSC, due to the ease of accessibility and differentiation potential. For this study, we worked with the St. Louis Cord Blood Bank, who had previously isolated UMSC from donor umbilical cords [46]. Given the heterogeneity of isolated MSC cultures, we looked at the cell surface composition to confirm the presence of MSC within our isolates. We found that the isolated UMSC showed the markers CD90, CD73, and CD105 which are the accepted markers of mesenchymal stem cells derived from various tissues, including umbilical tissue [50, 60]. However, mesenchymal stem cells are also described as lacking a number of other markers, such as CD11b, CD14, CD34, or CD45 [50]. We have demonstrated that our UMSC lack the markers CD14, CD34, or CD45. In addition, they lacked the marker CD20, a marker of mature B cells [61], which would indicate a hematopoietic lineage. Another requirement of MSC is the ability to show trilineage mesodermal differentiation potential (adipogenic, chondrogenic, and osteogenic) [50], which was demonstrated previously in the UMSCs used in this study [46]. Therefore, we concluded that our UMSC culture is a population of stem cell of non-hematopoietic origin.

Treatment of Morquio A deficient cells with GALNS enzyme involves uptake of free-available GALNS. This uptake is facilitated by the M6P receptor [1], a common receptor target for lysosomal proteins [59, 62]. We tested the hypothesis that UMSC follows this same pathway by treating UMSC with GALNS in the presence and absence of M6P. The UMSC lysates showed an increase in activity from 5 to 24 h of culture with GALNS in solution, which indicated uptake of the enzyme over time. This uptake was competitively inhibited by 95% in the presence of M6P, indicating that UMSC uptakes GALNS exclusively through the M6P receptor. Previous studies have demonstrated M6P-mediated inhibition of GALNS ranging from 90 to 99% [1, 63]. There is evidence of other enzymes being released from stem cells [64, 65], with one study suggesting release from UMSC [44], so we tested UMSC-conditioned media for any indication of GALNS release. We found trace amounts of active GALNS in UMSC-conditioned media, which was more prominent after concentrating. This confirmed that active GALNS is among the releasate of UMSC. These results provide direct evidence of the enzyme presence in the releasate rather than uncharacterized content release [44].

With evidence that UMSC released active GALNS, we tested whether deficient cells would benefit from this release. For this purpose, we grew deficient cells in the presence of UMSC. Initially, there was no increase, but over time GALNS activity in the deficient fibroblasts improved in the presence of UMSC. This suggests that the GALNS-containing releasate from UMSC was the source of GALNS enzyme that resulted in increased GALNS activity within deficient fibroblasts. To understand whether there was any evidence of active enzyme transported from UMSCs within organelles, we labeled lysosomes within UMSC and co-cultured them with deficient Morquio A fibroblasts. We observed that over time these UMSC-derived organelles emerged within the deficient cells. This signal from the organelles appeared to fade rapidly (after 20 min). We attribute this to photobleaching of the dye, rather than to the actual loss of the organelles from the deficient cells, which we observed happened quickly during our initial imaging of the feeder cells.

Organelle transport has been studied in the past. However, the vast majority of this documented transport has been observed through the use of tunneling nanotubes. Many of these studies focus on the transport of mitochondria from MSC [66–68]. The transfer of lysosomes though tunneling nanotubes has been demonstrated in macrophages but not in MSCs [69]. In our study, we did not investigate the possible formation of tunneling nanotubes due to the co-culture methods used since they utilize physical barriers between cell populations, which prevent this form of cell communication.

A significant portion of recent MSC therapy research has revolved around the ability of MSC to release EVs [70–72]. Our experiments have thus far shown that UMSC secretes active GALNS and that deficient cells can uptake the released enzyme in vitro. After observing the transfer of organelles from UMSC to deficient cells, we suspected that their release might be facilitated through EVs. Using differential ultracentrifugation [54], we isolated the EVs released from UMSC. Earlier studies have shown that these MSC-derived EVs contain a variety of molecules including growth factors, nucleic acids, microRNAs, and enzymes, which are enveloped by the membrane of these vesicles [73–76]. We found that suspending the EVs in lytic solutions or sonicating them would result in higher enzyme activity than those suspended in PBS. From there, we concluded that the low activity of non-lysed vesicles was due to the EVs encapsulating the enzyme, restricting its access to substrate when assaying.

We also wanted to characterize the composition of the EVs released by UMSC. While many publications described the vesicles as exosomes [36, 37], we have referred to the EVs isolated as a mixture of exosomes and microvesicles [54]. Not only do microvesicles and exosomes vary in size, but they are also formed in different locations within the cell. Since exosomes are formed within multivesicular endosomes [34] and microvesicles are formed by the budding and shedding of the plasma membrane [35, 77], it stands reason that these vesicles would display different sets of markers on their surfaces. The surface of both types of EVs display different tetraspanins, with exosomes displaying CD53, CD9, and CD151 and microvesicles displaying CD9, CD14, LFA1 (CD11a), and PECAM1 [35]. By staining our EV isolate and performing FACS sorting, we could determine that our isolate contains a mixture of both exosomes and microvesicles. The strongest response was CD9 staining, which is present in both populations. One of the major challenges of this experiment was the low incident number. Further experimentation will be to determine the quantitative ratio of exosomes to microvesicles, as well as determine which vesicle best serves as the vehicle for GALNS delivery.

After demonstrating that UMSC were capable of releasing GALNS-containing EVs, we next needed to show that they could facilitate uptake by deficient cells. While a complete picture of the mechanisms of EV uptake is not known, some pathways have been discovered. In most cases, uptake is shown to occur through endocytosis, but the mechanisms of endocytosis are not universal [78, 79]. For example, some cases of exosome uptake have been shown to occur via the Syndecan-syntenin-ALIX pathway [80]. Thus, we suspected that uptake might be through a different mechanism when compared to freely available GALNS. To test this, we repeated our M6P inhibition experiment using deficient fibroblasts and varying doses of GALNS or EVs. While we found that a 2000U dose of GALNS was the most effective dose, this improvement in fibroblasts was short-lived and began declining after 48 h. When treating with isolated EVs or a comparable dose of freely available GALNS, we noticed an improvement that, while low, was more consistent over the 48-h period. In addition, we did not observe significant changes of enzyme activity or inhibition after the addition of M6P. The full picture of EV uptake is far from complete. In addition to the mechanisms mentioned earlier, other mechanisms of uptake include clathrin-independent phagocytosis mediated by Flotillin-1 [81], phagocytosis [82], and even fusion with target cell membrane [83]. Our results eliminate one potential pathway of uptake, but further research is needed to elucidate the method of EVs uptake by Morquio A deficient fibroblasts.

The amount of GALNS present in our EV isolates has been extremely low. Further experiments to test the efficacy of GALNS uptake would require EVs with higher GALNS content. To accomplish this, we transformed a line of UMSC to constitutively express the GALNS enzyme. Similar techniques had been used previously, such as overexpressing erythropoietin to treat ischemia. However, this transfection was not stable [84]. Transfection of MSC, particularly stable, non-viral transfection, has not yet been perfected. Additionally, this transfection loses efficacy in vitro over repeated passages of MSC culture [85]. Finally, transfection by lipofection can be very inconsistent, and one group can demonstrate 20% efficacy [85], while another can only transform 7% under the same conditions [86]. We found two clones that have over 200% higher enzyme activity offering a promising treatment approach. Another strategy to increase enzyme activity that will be explored in the future is to isolate EVs from unmodified UMSC and use sonication or permeabilization to enrich the EVs with GALNS [87].

As non-embryonic stem cells, a major challenge when working with MSC is the loss of their “stemness” over time, a trait which is largely dependent on the tissue of origin [88–90]. While UMSC maintain their qualities longer than those isolated from other tissues [91], the effect is not indefinite. When we compared activity of EVs isolated from early-passage (p6 or earlier) to later passages (passages after p10) UMSC, we found that late-passage continued to produce GALNS-containing EVs. This continued functionality was consistent with previous findings [46, 92]. Further in vitro experimentation would need to be conducted to determine when the loss of this capability occurs.

## Conclusions

In conclusion, UMSC can deliver functional GALNS enzyme to deficient cells in vitro. This delivery, facilitated by EVs, presents a novel method for reducing the accumulation of GAGs within these cells. EVs contain many components, including active GALNS, which can be uptaken and subsequently used in deficient cells. This uptake process is not dependent on the presence of the UMSC, and unlike current treatments is not reliant on the M6P receptor. Further research is needed to determine the optimal methods of EV enrichment for treatment, as well as expansion of treatments into an in vivo model.

## Data Availability

All data generated or analyzed during this study are included in this published article.

## Abbreviations

GALNS: *N*-acetylgalactosamine-6-sulfate sulfatase
GAGs: Glycosaminoglycans
ERT: Enzyme replacement therapy
UMSC: Umbilical mesenchymal stem cells
EVs: Extracellular vesicles
KS: Keratan sulfate
C6S: Chondroitin-6-sulfate
rhGALNS: Recombinant human *N*-acetylgalactosamine-6-sulfate sulfatase
M6P: Mannose-6-phosphate
GUSB: β-Glucuronidase
